# A statistical model for mapping morphological shape

**DOI:** 10.1186/1742-4682-7-28

**Published:** 2010-07-01

**Authors:** Guifang Fu, Arthur Berg, Kiranmoy Das, Jiahan Li, Runze Li, Rongling Wu

**Affiliations:** 1 Department of Statistics, Pennsylvania State University, University Park, PA 16802, USA; 2 Center for Statistical Genetics, Pennsylvania State University, Hershey, PA 10733, USA; 3 Center for Computational Biology, Beijing Forestry University, Beijing 100083, China

## Abstract

**Background:**

Living things come in all shapes and sizes, from bacteria, plants, and animals to humans. Knowledge about the genetic mechanisms for biological shape has far-reaching implications for a range spectrum of scientific disciplines including anthropology, agriculture, developmental biology, evolution and biomedicine.

**Results:**

We derived a statistical model for mapping specific genes or quantitative trait loci (QTLs) that control morphological shape. The model was formulated within the mixture framework, in which different types of shape are thought to result from genotypic discrepancies at a QTL. The EM algorithm was implemented to estimate QTL genotype-specific shapes based on a shape correspondence analysis. Computer simulation was used to investigate the statistical property of the model.

**Conclusion:**

By identifying specific QTLs for morphological shape, the model developed will help to ask, disseminate and address many major integrative biological and genetic questions and challenges in the genetic control of biological shape and function.

## Background

Morphological shape is one of the most conspicuous aspects of an organism's phenotype and provides an intricate link between biological structure and function in changing environments [1,2]. For this reason, comparing the anatomical and shape feature of organisms has been a central element of biology for centuries. Nowadays, attempts have been made to unlock the genetic secrets behind phenotypic differentiation in developmental shape [3], understand the origin and pattern of shape variation from a developmental perspective [4,5], and predict the adaptation of morphological shapes in a range of environmental conditions [6].

Three major advances in life and physical science during the last decades will make it possible to study shape variation and its biological underpinnings. First, DNA-based molecular markers allow the identification of quantitative trait loci (QTLs) and biochemical pathways that contribute to quantitatively inherited traits such as shape. In his seminal review, Tanksley [3] summarized some major discoveries of genes for fruit size and shape in tomato. In a long process of domestication, tremendous shape variation has occurred in tomato fruit from almost invariably round (wild or semiwild types) to round, oblate, pear-shaped, torpedo-shaped, and bell pepper-shaped (cultivated types). Some of the QTLs that cause these differences, namely *fw2.2*, *ovate*, and *sun*, have been cloned [7-9].

Second, digital technologies through computerized analyses and processing procedures can obtain a comprehensive representation of the involved objects, capable not only of representing most of the original information, but also of emphasizing their less redundant portions [10-15]. Third, statistical and computational technologies have well been developed for analyzing high-dimensional, large-scale, high-throughput data of high complexity [16,17]. With the development of missing data analysis, Lander and Botstein [18] have been able to pioneer an approach for dissecting complex quantitative traits into individual QTLs using genetic linkage maps constructed with molecular markers. There has been a vast wealth of literature in the development of QTL mapping models (see [19-25] among many others).

The motivation of this study is to develop a statistical and computational model for mapping specific QTLs that are responsible for differences in morphological shape. Historically, genetic mapping has been focused on the genetic control of a trait at a static point, ignoring the dynamic behavior and spatial properties of the trait. Now, by integrating the developmental principle of trait growth, a new genetic mapping approach, called functional mapping [26-28], can be used to study the dynamic control of genes in time course. The central idea of functional mapping is to connect the genetic control of a developmental trait at different time points through robust mathematical and statistical equations. Complementary to functional mapping, the model developed for shape mapping in this study links gene action with key morphometric parameters of a shape within a statistical framework. We will perform computer simulation to examine the statistical properties of the model.

## Model

### Genetic Design

**Figure 1:**
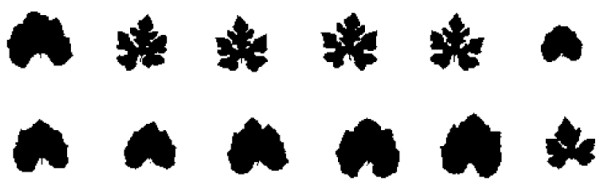
**The diagram of twelve leaf shapes from the backcross population**. Five of them are wild Cucurbita argyrosperma sororia and seven of them are cultivated cucurbita argyrosperma.

We assume a backcross design although the model can be modified to accommodate any other mapping designs. Consider a backcross progeny population of size *n*, founded with two inbred lines that are sharply contrasting in leaf shape. Because of gene segregation, there is a range of variation in leaf shape among the backcross progeny. Such shape variation is illustrated in Fig. 1 by using leaf morphology in cucurbit plants [29]. To map the shape trait, the mapping population is typed for a panel of molecular markers from which a genetic linkage map covering the genome is constructed. The statistical approach for linkage analysis and map construction is reviewed in Wu et al. [30]. Assume that there are some specific QTLs responsible for the biological shape. The approach being developed aims to detect and map such QTLs by capitalizing on knowledge about shape analysis and biological principles behind shape formation and variation.

### Shape Analysis

According to the definition of Kendall [31], "shape is all the geometrical information that remains when location, scale and rotational effects are filtered out from an object". Assume that each backcross progeny is measured for the leaf shape as shown in Fig. 1. For a given shape, *I**^i ^***(*i *= 1, ..., *n*), described by a black and white image, it is gridded as an *L *× *L *matrix, where L is the number of pixels in the row and column. At each point in the matrix, we use 0 to denote the background (black) and 1 to denote the leaf (including an arbitrary shape of it) (white). The 1/0 value of the matrix is assumed to follow a Bernoulli distribution. All these *n *shapes, *T = *{*I*^1^, *I*^2^, ..., *I*^n^}, need to be aligned, in order to minimize the interference caused by pose variations. This can be carried out by establishing a coordinate reference with respect to position, scale and rotation, commonly known as pose to which all shapes are aligned [10,12,14]. Denote the pose parameter for each shape *I**^i ^**by p**^i ^**= [a, b, h, θ]**^T ^***where *a *and *b *correspond to *x *and *y *translations, *h *is the scaling parameter, and *θ *corresponds to rotation. The transformed image of *I**^i^***, based on the pose parameter *p**^i^***, is denoted by *Ĩ^i^*, defined as

where

which yields(1)

The translation matrix T [*p*] is the product of three matrices: a translation matrix *M*(*a*, *b*), a scaling matrix *H*(*h*), and an in-plane rotation matrix *R*(*θ*). The transformation matrix *T *[*p*] maps the coordinates (*x, y*) ∈ *R*^2 ^into coordinates  ∈ *R*^2^, where *x, y *= 1, ..., *L*.

An effective strategy to jointly align the *n *binary images is to use a gradient descent to minimize the following energy function:(2)

where Ω denotes the image domain. Minimizing the energy function (2) is equivalent to simultaneously minimizing the difference between any pair of binary images in the training database. What we would like to estimate is the pose parameter *p**^i ^***for each *I**^i^***.

The derivative respective to *p**^i ^***of equation (2) is(3)

By a chain rule and equation (1), we get

**Figure 2:**
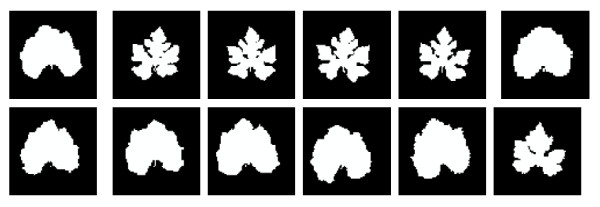
**Leaf shapes after alignment for leaf shapes shown in Fig. 1**.

Hence, we can obtain the value of  E as long as *p^i ^*and *Ĩ^i ^*are given in each iterative step. The steepest gradient algorithm is then used to minimize *E *in (2) and get the pose parameter *p^i ^*for each shape *I^i^*. All the training shapes after the alignment procedure described above are obtained (see Fig. 2).

### Statistical Model

After all the training shapes are aligned, a shape representation scheme needs to be chosen for T = { *Ĩ*^1^, *Ĩ*^2^, ..., *Ĩ*^n^}., i.e., the transformed images, which now become continuous variables. The signed distance function was used as a shape descriptor to represent the contours of the shape. Each contour is embedded as the zero level set of a signed distance function with negative distances assigned to the inside and positive distances assigned to the outside. This technique yields *n *level sets functions *Y = *{*Y_1_*, *Y_2_*, ... *Y_n_*} corresponding to above *n *aligned training shapes. From the standpoint of QTL mapping, we treat *Y = *{*Y_1_*, *Y_2_*, ..., *Y_n_*} as the multiple phenotypic traits of *n *individuals. For a progeny *i *(*i *= 1, 2, ..., *n*), we have(4)

Thus, each individual has a total of *m *= *L*^2 ^phenotypes.

For the backcross progeny population, there are always two different genotypes at each locus. The genotypes at a shape QTL, expressed as *QQ *(denoted as 1) and *Qq *(denoted as 2), cannot be observed directly but can be inferred from the markers that are linked to the QTL. For this reason, the basic statistical model for QTL mapping is based on a mixture model, in which each observation *Y *is assumed to have arisen from one of the two groups of QTL genotypes, each group being modeled from a density function (frequently a normal distribution is assumed). Thus, the population density function of *Y *is(5)

where *ω *represents the mixture proportions (*ω*_1|*i*_, *ω*_2|*i*_), which are constrained to be nonnegative and sum to unity, *ϕ_j _*is the expectation parameter specific to different QTL genotypes *j *= 1, 2, and *η *is the variance-covariance parameter common to all genotype groups, and *f_j_*(*Y_i_*|*ϕ_j_*,*η*) is the probability density function for QTL genotype *j*. After images are transformed, Y*_i_* can be assumed to follow a multivariable normal distribution, i.e.,(6)

with the expectation matrix of each QTL genotype expressed as(7)

and (*m × m*) residual variance-covariance matrix of the variables ∑. If some patterns exist, we will use *ϕ_j _*to model the mean structure of *μ_j _*and *η *to model the covariance structure of ∑.

In order to simplify the problem, we use the most natural sampling strategy to utilize the *L × L *rectangular grid of the training shapes to generate *m = L × L *lexicographically ordered samples (where the columns of the matrix grid are sequentially stacked on top of one other to form one large row). Also, we assume that all the observations in the long row are independent among the progeny. Now, from equation (5), we get the likelihood function as(8)

where the mean matrix of QTL genotype *j (μ_j_*) is modeled by parameter *ϕ_j_*, and covariance matrix (∑) modeled by parameter *η*.

### Computational Algorithm

To obtain the maximum likelihood estimates (MLEs) of parameters in likelihood (8), we implement a standard EM algorithm. In the E step, we compute the posterior probability with which a backcross individual carries a QTL genotype *j *using(9)

In the M step, we estimate the parameters using(10)

for *j *= 1, 2 and *k *= 1, 2, ..., *m*.

The EM steps are iterated between equations (9) and (10) until the estimates converge to stable values. It should be pointed out that the data set for shape analysis is highly sparse and high-dimensional. For example, if a shape is described by (256 × 256) pixels, i.e., L = 256, then we will have m = 256^2 ^= 65, 536, and an (*n *× 65, 536) matrix for the phenotypic observations. Several approaches will be developed to model the structure of the variance-covariance matrix. One of the simplest approaches is to use . This choice is large enough to assure that various levels of differences lie well within a Gaussian distribution.

### Hypothesis Tests

A hypothesis about the existence of a significant QTL that controls a morphological shape can be tested by calculating the log-likelihood ratio under the hypotheses:(11)

As like an usual mapping approach, shape mapping has a problem of uncertain distribution for the log-likelihood test statistic. However, an empirical approach based on permutation tests, which does not rely on the distribution of log-likelihood ratios, can be used to determine the threshold for claiming the existence of a significant QTL.

## Computer Simulation

Cucurbit (*Cucurbita argyrosperm*) plants display tremendous variation in leaf shape between cultivars and wild types [29]. By mimicking leaf morphologies of this species, we performed simulation studies to examine the statistical behavior of our shape mapping model. A backcross population of 200 progeny was simulated for a linkage group with 11 equally spaced markers. A QTL that determines leaf shape is hypothesized on the third marker interval. The phenotypic values of the shape were simulated with a (75 × 75) dimension by *Y_i _*= *ξ_i_μ*_1 _+ (1-*ξ_i_*)*μ*_2 _+ *e_i_*, where *μ_j _*is the mean shape matrix for QTL genotype *j *(*j *= 1, 2), *ξ_i _*is the indicator variable defined as 1 and 0 if progeny *i *carries QTL genotype *QQ *(1) and *qq *(2), respectively, and *e_i _*follows a multivariate normal distribution with mean vector zero and covariance matrix ∑. To simplify computing, we assumed that ∑ is an identity matrix. We designed two simulation schemes to test our shape mapping algorithm.

**Figure 3:**
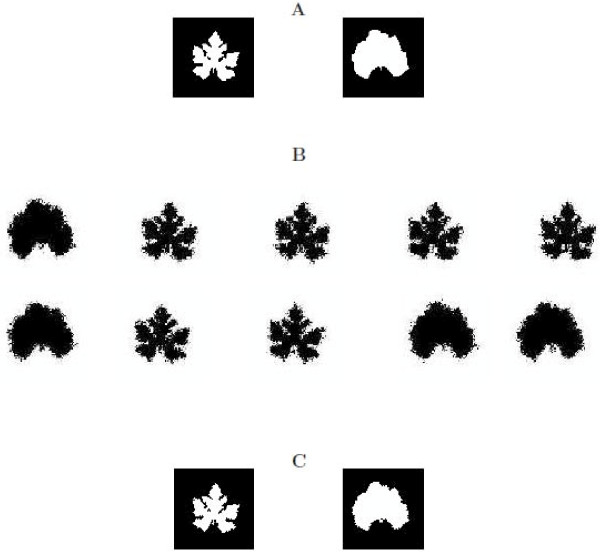
**The first simulation scheme: A "big" QTL controls differences in leaf shape between wild types and cultivars for cucurbit plants**. **A**: Two given QTL genotypes, *QQ *for the wild type (left) and *Qq *for the cultivar (right); **B**: Part of the simulated backcross progeny; **C**: Two estimated QTL genotypes, *QQ *for the wild type (left) and *Qq *for the cultivar (right).

The first scheme assumes that there exists a "big" QTL which triggers a tremendous effect on the difference in leaf shape of cucurbit plants between their cultivars and wild types. This QTL has two different genotypes, one, *QQ*, corresponding to the wild type shape (right) and the second, *Qq*, to the domesticated shape (left) (Figure 3A). The QTL genotypes are determined by the conditional probability of a QTL genotype, conditional upon the genotypes of the two markers that flank the QTL (see [30]). Part of the 200 progeny simulated with two assumed QTL genotypes were given in Figure 3B, in which some leaf shape looks more like the wild type, some more like the domesticated type, and the other is in between. The model described above was used to analyze the simulated data. The log-likelihood ratio test statistic calculated under hypotheses (11) is greater than the critical threshold for testing the existence of a QTL obtained from permutation tests, suggesting that two genotype-specific shapes for *QQ *and *Qq *were detected and identified. Figure 3B also illustrates the shapes of two detected QTL genotypes from the simulated data. As shown, the estimated shapes are similar to the true shapes for the two backcross QTL genotypes, suggesting that our model has great power to identify the QTL that control morphological shape.

**Figure 4:**
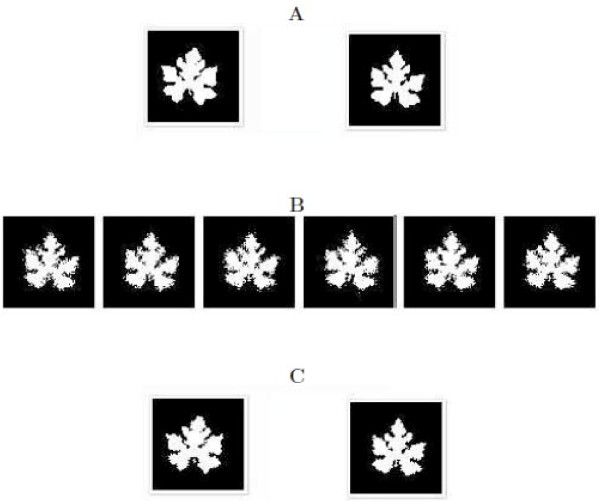
**The second simulation scheme: A "small" QTL controls differences in leaf shape among different plants from wild types of cucurbit plants**. **A**: Two given QTL genotypes, *QQ *for the wild type (left) and *Qq *for the cultivar (right); **B**: Part of the simulated back-cross progeny; **C**: Two estimated QTL genotypes, *QQ *for the wild type (left) and *Qq *for the cultivar (right).

**Figure 5:**
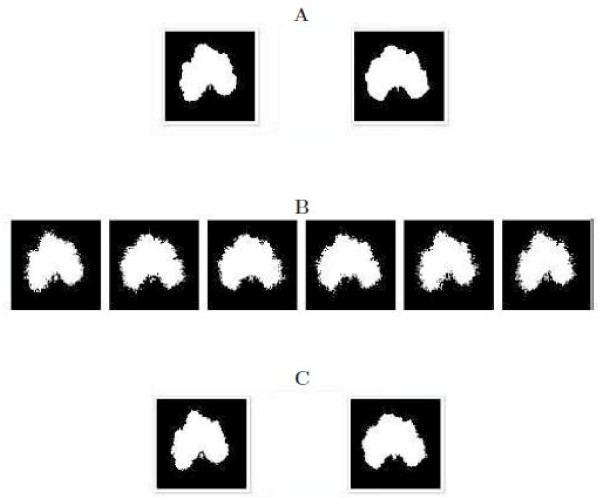
**The second simulation scheme: A "small" QTL controls differences in leaf shape among different plants from cultivars of cucurbit plants**. **A**: Two given QTL genotypes, *QQ *for the wild type (left) and *Qq *for the cultivar (right); **B**: Part of the simulated backcross progeny; **C**: Two estimated QTL genotypes, *QQ *for the wild type (left) and *Qq *for the cultivar (right).

The second scheme simulated two QTLs that determine the differences of leaf shape among wild-type plants and domesticated plants, respectively. Compared to the "big" QTL assumed in the first scheme, these two QTLs are "small" because their two genotypes correspond to slightly different leaf shapes. Figures 4 and 5 provide the results about shape mapping for wild-type plants and domesticated plants, respectively. In the upper panel (A) of each figure, two original QTL genotypes are assumed, from which 200 backcross progeny were simulated with a range of leaf shape. The middle panel (B) gives part of the backcross. In the bottom panel (C), two genotypes were estimated using our algorithm. It can be seen that the model can well detect a QTL even if it has a small effect on morphological shape.

**Figure 6:**
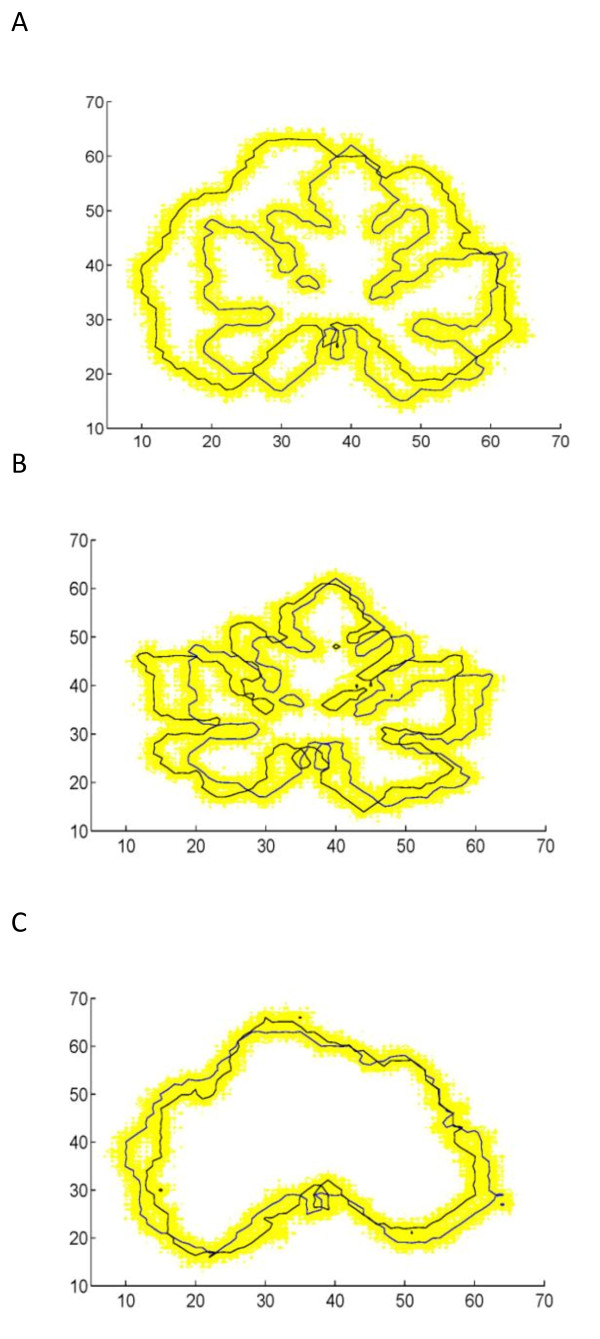
**The fitness of estimated QTL genotypes to simulated leaf shape in a backcross**. **A**: A "big" QTL for the shape difference between wild types and cultivars of cucurbit plants. **B**: A "small" QTL for the shape difference between different wild types. **C**: A "small" QTL for the shape difference between different cultivars.

**Figure 7:**
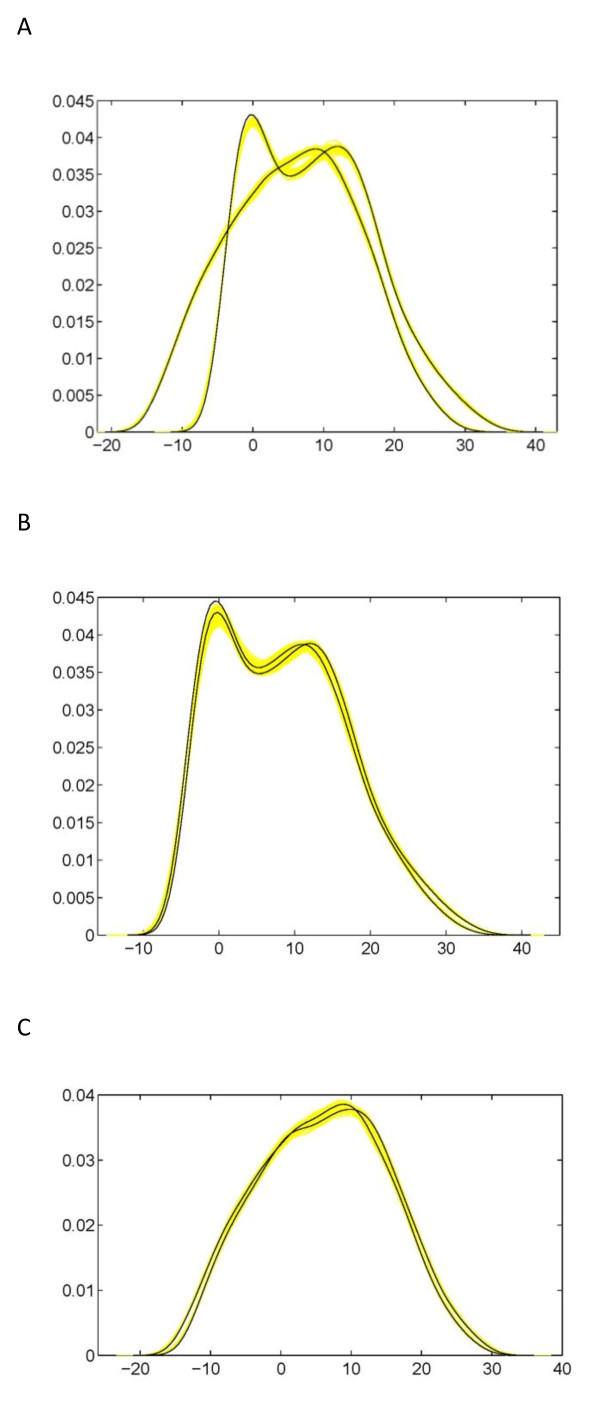
**Density functions of leaf shape for the simulated backcross (yellow) and two QTL genotypes**. **A**: A "big" QTL for the shape difference between wild types and cultivars of cucurbit plants. **B**: A "small" QTL for the shape difference between different wild types. **C**: A "small" QTL for the shape difference between different cultivars.

To show the fitness of our model, we put the estimated QTL genotypes on the simulated backcross population for the first (A) and second (B and C) simulation scheme (Fig. 6). The leaf shape of two QTL genotypes in each case well covers the simulated leaf shape, showing a good fitness of the mapping model. Also, we calculated the density functions for each simulated progeny and two QTL genotypes for each simulation scheme (Fig. 7). The "big" QTL displays two distinct modes of distribution (Fig. 7A), whereas there is a small difference in the density functions of two genotypes for each of two "small" QTLs (Fig. 7B,C). By comparing Fig. 1A with Fig. 7B and 7C, we can obtain the basic information about how well different QTL genotypes are separated when QTLs exert different effects on leaf shape.

## Discussion

When specific genes that control morphological shape and physiological function are identified, we are in an excellent position to address fundamental questions related to growth, development, adaptation, domestication, and human health. In the past decades, the increasing availability of DNA-based markers has inspired our hope to map genes or quantitative trait loci (QTLs) for complex phenotypes [19-25]. However, only several studies have been alert to map so-called shape genes; a few successful examples are the positional cloning of genes for fruit shape in tomato [3,7-9]. These successes result from the fact that a major mutation occurs to determine shape difference. For many quantitatively inherited shape traits, genetic mapping will provide a powerful tool for characterizing QTLs affecting morphological shape. Klingenberg and colleagues [4,5] have developed quantitative genetic theory to estimate the heritability of shape by integrating geometric shape analysis. This theory was used to map specific QTLs for morphometric shapes in the mouse [32,33]. Airey et al. [34] used Procrustes superimposition to study shape differences in the cortical area map of inbred mice.

In this article, we present a new statistical model for mapping shape QTLs in a segregating population. The new model embeds shape analysis within a mixture model framework in which different types of morphological shape are defined for individual genotypes at a QTL. The model was solved using a traditional shape correspondence analysis approach and EM algorithm. The advantage of shape mapping lies in its capacity to quantify subtle differences in any corner of a morphological shape and detect specific QTLs that contribute to these differences. Results from simulation studies suggest that the model has reasonably high power to detect a QTL that control shape difference. Even with a modest sample size (200), the model is able to discern the effect of a QTL with a small effect on morphological shape. The model can be easily extended to model epistatic interactions on morphological shape by including more components in the mixture model.

The model will be needed to be modified for integrating developmental events and their consequences into ontogenetic trajectories of shape. Modern biological studies display an increasing interest in understanding shape variation in ontogenetic processes that bring about differentiation at an adult stage [35-37]. In a longitudinal study of radiographs of the Denver Growth Study, Bulygina et al. [37] investigated the morphological development of individual differences in the anterior neurocranium, face, and basicranium. The modified model can map the QTLs that cause variation in shape developmental trajectories.

In biology, a cell or organ fulfill certain biological functions through its shape. Shape is thought to govern the extent and pattern of energy, matter and signal transduction through the surface and inner structure of the biological object. For this reason, an understanding of biological curvature and texture has received a surge of interest in structural biology. The new model can be extended to map the QTLs that determine a three-dimensional (3D) shape and texture of a biological object. Vision technologies have been developed to estimate the 3 D shape of an object from 2 D image data without information about its texture (albedo), its pose and the illumination environment [38,39]. These technologies include a 3 D morphable model (3DMM) that represents the 3 D shapes and textures as a linear combination of shapes and textures principal components, a stochastic Newton optimization algorithm that ts the 3DMM to a single facial image, thereby estimating the 3 D shape, the texture and the imaging conditions, and a multi-features fitting algorithm that uses not only the pixel intensity but also other image cues such as the edges and the specular highlights. Statistical models can be developed to map QTLs that control the 3 D shape and texture of a biological object with image data. A series of hypothesis tests about the genetic control of topological features (such as stepness and ridgeness) and texture of a shape will be formulated.

## Competing interests

The authors declare that they have no competing interests.

## Authors' contributions

GF derived the model and performed simulation studies. AB, KD, and JL participated in simulation studies. RL participated in the design of the study. RW conceived of the study, coordinated the design and simulation studies, and wrote the manuscript. All authors read and approved the final manuscript.
